# Pharmaceutical development and optimization of azithromycin suppository for paediatric use

**DOI:** 10.1016/j.ijpharm.2012.11.040

**Published:** 2013-01-30

**Authors:** Tina Kauss, Alexandra Gaubert, Chantal Boyer, Boubakar B. Ba, Muriel Manse, Stephane Massip, Jean-Michel Léger, Fawaz Fawaz, Martine Lembege, Jean-Michel Boiron, Xavier Lafarge, Niklas Lindegardh, Nicholas J. White, Piero Olliaro, Pascal Millet, Karen Gaudin

**Affiliations:** a Univ. Bordeaux, EA 4575 Analytical and Pharmaceutical Developments Applied to Neglected Diseases and Counterfeits, Bordeaux, France; b Univ. Bordeaux, Laboratory of Galenic Pharmacy and Biopharmacy, Bordeaux, France; c Univ. Bordeaux, FRE 3396 CNRS Pharmacochimie, Bordeaux, France; d Univ. Bordeaux, Laboratory of Organic and Therapeutic Chemistry, Pharmacochimie, Bordeaux, France; e EFS (Etablissement Français du Sang) Aquitaine-Limousin, Place Amélie Rabat-Léon, BP 24, 33035 Bordeaux, France; f Tropical Medicine, Mahidol University, 420/6 Rayvithi Road, Bangkok 10400, Thailand; g Centre for Tropical Medicine, Nuffield Department of Medicine, University of Oxford, UK; h UNICEF/UNDP/WB/WHO Special Program for Research & Training in Tropical Diseases (TDR), Geneva, Switzerland

**Keywords:** Azithromycin, Antibiotic, Rectal, Suppository, Pharmaceutical development, Paediatric solid dispersion

## Abstract

Pharmaceutical development and manufacturing process optimization work was undertaken in order to propose a potential paediatric rectal formulation of azithromycin as an alternative to existing oral or injectable formulations. The target product profile was to be easy-to-use, cheap and stable in tropical conditions, with bioavailability comparable to oral forms, rapidly achieving and maintaining bactericidal concentrations. PEG solid solution suppositories were characterized *in vitro* using visual, HPLC, DSC, FTIR and XRD analyses. *In vitro* drug release and *in vivo* bioavailability were assessed; a study in rabbits compared the bioavailability of the optimized solid solution suppository to rectal solution and intra-venous product (as reference) and to the previous, non-optimized formulation (suspended azithromycin suppository). The bioavailability of azithromycin administered as solid solution suppositories relative to intra-venous was 43%, which compared well to the target of 38% (oral product in humans). The results of 3-month preliminary stability and feasibility studies were consistent with industrial production scale-up. This product has potential both as a classical antibiotic and as a product for use in severely ill children in rural areas. Industrial partners for further development are being sought.

## Graphical abstract

**Figure:**
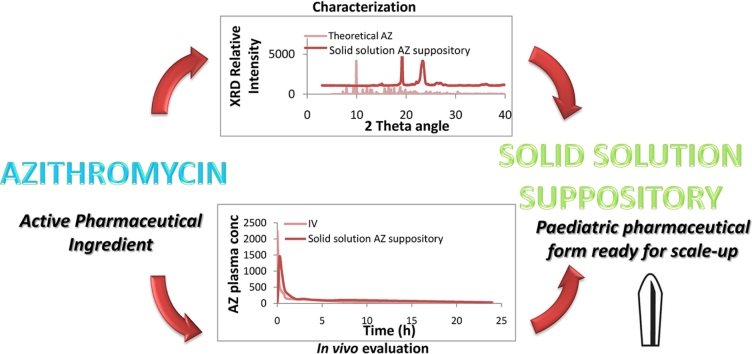

## 1 Introduction

Bacterial infections still take a heavy morbidity and mortality toll on the lives of children, particularly those under 5 years of age (WHO, 2012). Against this scenario, there is a lack of paediatric formulations of antibiotics that are adapted to the needs of the developing world, where these infections are mostly prevalent. Particularly needed are formulations that can be administered by unqualified personnel to children who cannot take oral medications (“non-per-os”) because their conditions are deteriorating.

The desired target product profile (TPP) was: (i) an antibiotic with a spectrum of action covering the main agents causing paediatric infections; (ii) use in both uncomplicated and complicated cases (where oral administration is not possible (patient “non-per-os”)); (iii) safe and easy to use by untrained personnel; amenable to near-home use; (iv) cheap; (v) stable in tropical conditions of temperature and humidity.

Work conducted in our laboratory (Kauss et al., 2012) had investigated options for a paediatric rectal formulation of azithromycin (AZ). The rectal route is acceptable in the targeted countries (Simba et al., 2009, Thera et al., 2007), and can be used in both uncomplicated and complicated (“non-per-os” cases); there is evidence, for instance, that rectal artesunate can save lives in case of malaria (Gomes et al., 2009). AZ (a macrolide) was considered as a drug candidate for this indication because of its broad antibacterial spectrum of activity and pharmacokinetic properties (distribution and concentration in infected organs, prolonged half-life offering the convenience of once daily administration) (Jacobs et al., 2005, Langtry and Balfour, 1998, Van Bambeke and Tulkens, 2001). Among existing chemical forms of AZ, the AZ dihydrate was chosen because of its stability (Gandhi et al., 2002).

There is no rectal formulation of a macrolide on the market, and little information exists on their rectal availability. The reported rectal bioavailability would be acceptable for erythromycin (28–54% bioavailability, varying with age (Stratchunsky et al., 1991)) but very low for azithromycin (3.2% bioavailability (Bergogne-Bérézin and Bryskier, 1999)).

In order to improve AZ bioavailability (Kauss et al., 2012) we tried: (i) enhancing viscosity and muco-adhesiveness to prolong the residence time with rectal gels, (ii) dry form formulated as rectal capsule, and (iii) enhancing solubility with solid dispersion suppositories. Fatty-base suppositories were excluded because their low melting point makes them incompatible with tropical conditions. The most promising prototype was the suspended AZ PEG suppositories based on solid dispersion approach, which attained 28% bioavailability (Kauss et al., 2012). In a solid dispersion, active ingredients are dispersed in an inert carrier or matrix of solid state, prepared by melting, solvent or melting-solvent method (Chiou and Riegelman, 1971). Solid dispersions include eutectic mixtures, solid solutions, glass solutions and suspensions, amorphous precipitations, compound or complex formation, combinations and miscellaneous mechanisms (Chiou and Riegelman, 1971).

The aim of the current study was to optimize the AZ suppository formulation in order to enhance its rectal bioavailability and render it amenable to further development.

## 2 Materials and methods

### 2.1 Materials

Azithromycin dihydrate (AZ) was generously donated by Pfizer (France). Zithromax^®^ (Pfizer, USA) was used as IV AZ formulation.

Pharmaceutical excipients, namely polyethylene glycol (PEG) 1500 and 4000 purchased from Fagron (Spain) and Miglyol 812N (Inresa, France) were of pharmaceutical grade. All other chemicals and solvents were of analytical reagent grade. Water was purified and deionized by the Millipore^®^ Simplicity system (USA).

### 2.2 Suppository preparation

All the tested formulations except IV (Zithromax^®^, Pfizer) were developed in our laboratory.

All suppositories were prepared using the moulding method.

To obtain suspended AZ suppositories, PEGs were melted at 65 °C in a water bath and then AZ was dispersed under mechanical stirring at 150 rpm.

For co-melted AZ suppositories, PEGs were pre-melted at high temperature above the melting points of all constituents (130 °C) in an oven (Jouan Paris, France) and then AZ was added. The preparation was left in the oven until AZ was melted and stirred to obtain a homogeneous blend.

Finally, in the case of solid solution AZ suppositories, PEGs were melted at 90 °C in a water bath and AZ was added. The mixture was stirred until obtaining a homogenous limpid blend.

For all suppositories, the blend was cooled to a temperature of 55–60 °C before being poured in 2 g suppository moulds. They were allowed to harden at room temperature in a dessicator for at least 24 h and stored in individual alu/alu blister or kept in their plastic moulds in a fridge before further analysis or use.

While screening different options for the feasibility of solid solution suppositories, the AZ content was varied and changes compensated with the PEG mixture content (qsf 100%, while keeping the ratio PEG 1500/PEG 4000 constant). For manufacturing optimization, the manufacturing process was varied while keeping the formulation unchanged. For scale-up studies, batch quantities were increased while the process and the formulation remained the same.

### 2.3 *In vitro* pharmacotechnical controls of AZ PEG suppositories

Suppositories were visually evaluated at each withdrawal and in each storage condition; the colour, the limpidity and consistency were taken into account. The melted aspect of suppositories was also observed on a watch glass. For this purpose, crushed suppository aliquot of approximately 50 mg was melted at 70 °C in an oven (Jouan-Paris, France) on the watch glass.

Dissolution behaviour of AZ suppositories (*n* = 6 for each formulation and each storage condition) was compared using the Pharmacopoean II apparatus (SOTAX AT 7, Switzerland). Suppositories were introduced in bowls containing 250 ml of phosphate buffer (50 mM) pH 7.0 maintained at 37.0 °C ± 0.2 and 75 rpm. Samples (1 ml aliquot replaced by an equal volume of fresh dissolution medium) were withdrawn at time 0, 15, 30, 45, 60, 90 and 120 min using a 10 μm porous prefilter. Samples were diluted suitably using phosphate buffer before HPLC analysis.

AZ drug content was determined using HPLC method described beneath. A 65 mg of crushed suppository was dissolved in 20 ml with HPLC mobile phase. After 10 min of magnetical stirring, the filtered preparation was analyzed without further dilution.

### 2.4 HPLC analysis of azithromycin

AZ content and dissolution profile were determined by an HPLC system composed of 515 HPLC Pump Waters, Waters 2487 Dual *λ* Absorbance Detector and Waters 717 plus Autosampler (Waters, France). Data were managed using Millenium^32^ Chromatography manager (Waters, France). HPLC method was based on Gaudin et al. (2011) method. AZ was eluted using Luna C8 EC 5 μm, 150 mm × 4.6 mm column (Phenomenex, France) thermostated with Crococil oven (CIL, Saint Foy la Grande, France) at 45 °C. The mobile phase at apparent pH of 9 was composed by methanol/phosphate buffer 15 mM (80/20, v/v) at flow rate of 1.2 ml min^−1^. The injected volume was 10 μl and AZ was detected at 215 nm.

### 2.5 Differential scanning calorimetry (DSC) analysis

DSC analysis was performed using a differential scanning calorimeter (Mettler Toledo TA controller and DSC30, Switzerland) with STAR^e^ software. DSC method consisted in a heating rate of 5 °C min^−1^ in the range of 30–180 °C for thermograms, or 2 °C min^−1^ in the range of 30–150 °C for melting point determination (*n* = 3 for each formulation). Precisely weighted samples of 6–8 mg were in aluminium pans, sealed and perforated with a pin. An empty sealed perforated aluminium pan was used as reference. For comparison, theoretical melting points were calculated as a mean of individual melting points taking into account the relative content.

### 2.6 Crystallographic studies

Powder X-ray diffraction patterns for AZ, physical mixture, PEG 1500 and 4000, and AZ suppositories were acquired using a X-ray diffractometer (AXS D5005, Bruker, Germany). Powder samples were placed on a stainless steel support and exposed to Cu Kα radiation (*λ* = 1.54056 Å) in the range of 3° < 2*θ* < 40° in continuous scan mode using a step size of 0.02°, 2*θ* and a step time of 20 s. A generator tension of 30 kV and of 40 kV and current of 30 mA was used for the XRD analysis of AZ suppositories.

### 2.7 Infrared analysis

Spectra of AZ, PEG 1500 and 4000 and suppositories were analyzed by Fourier transformed infrared (FTIR), using Mattson model Genesis II FTIR spectrometer controlled by Winfirst software from Mattson instruments Inc. (Winlab. Instruments, Bagnolet, France). Samples were ground and mixed with potassium bromide at 1% weight ratio and then compressed at a pressure of 8 tonnes during 5 min (hydraulic press). Single beam spectra were recorded after averaging 20 scans at a resolution of 0.5 cm^−1^, between 4000 and 400 cm^−1^ and corrected against the background spectrum of the atmosphere. All spectra were obtained in the absorbance mode and then converted in transmittance. Calibration of the instrument was repeated periodically during operation.

### 2.8 Preliminary stability study

A preliminary stability study was realized on solid solution AZ suppository batch during three months. One part of the batch kept in individual alu/alu blister or plastic mould in a climatic chamber (Froilabo/Frilabo, France) was used for an accelerated degradation study (40 °C and 75% relative humidity) and the other one for an ambient condition stability study.

### 2.9 Animal pharmacokinetic experimentation

Animal experiments were performed at Etablissement Français du Sang (Aquitaine-Limousin, Bordeaux, France; accreditation number for animal experimentation no. A33063080).

Five adult healthy New-Zealand white rabbits, provided by “Le grand Claud” (Eyvirat, Dordogne, France), were used for the pharmacokinetic study of each form. Animals were placed into individual cages in controlled temperature (18–21 °C) and humidity (40–70%) room. They were fastened for 24 h prior and during the experimentation but allowed free access to water and glucose solution (5%).

All forms for *in vivo* evaluation in rabbits were prepared extemporaneously. Rectal formulation mass equivalent to 20 mg/kg was adjusted individually to each animal body weight. Solid solution suppositories were prepared as described in Section 2.2. Results from previously published work were used for suspended AZ suppository (Kauss et al., 2012, formulation D) for comparison. The rectal oily suspension was prepared by dispersion of AZ (sieved at < 250 μm) in medium chain triglyceride oil (Miglyol 812N) and served as rectal reference for PK studies. AZ IV solution used was the one commercially available (Zithromax^®^, Pfizer). An IV dose of 10 mg/kg was administered to animals.

The insertion into the animal's rectum was performed using 1 ml syringe for liquid formulations and 1 ml pipette like device with large aperture and piston for dry forms.

Blood samples (at least 1 ml of blood per time point) were collected into heparinized plastic tubes by inserting a 22 GA I.V. catheters (BD Insyte^®^, Spain) in peripheral ear vessels (opposite ear was used for IV administration). They were collected at pre-dose, then 12 samples in 48 h post-administration and kept on crushed ice before centrifugation at 1200 × *g* during 10 min within 30 min after collection. Only supernatants (at least 500 μl) were transferred into cryo tubes and conserved at −80 °C prior analysis.

The maximal concentration (*C*_max_), and the time to reach the maximal concentration (*T*_max_) of each formulation were taken directly from mean plasma concentration–time profile curves, whereas the area under the concentration–time curve (AUC) was calculated using trapezoidal rule. Standard deviations are given for all PK parameters. Absolute and relative bioavailability were estimated from mean AUC_0–24 h_ and AUC_0–48 h._ Data for 24 h were analyzed by comparison with previously obtained parameters for suspended AZ suppositories (Kauss et al., 2012).

### 2.10 Sample preparation and LC–MS/MS conditions for PK samples

Plasma samples underwent a solid-phase extraction (Oasis HLB, Waters, USA) before analysis by a LC–MS/MS system composed of a Hitachi LaChrom Elite LC (Hitachi, Japan) with an Esquier 4000 mass spectrometer equipped with an electrospray interface. Multiple reaction monitoring (MRM) was used during AZ quantification based on transitions *m*/*z* 749–591 and 753–595 for AZ and stable labelled AZ, respectively.

The separation was carried out on a Hypersil Gold CN 150 mm × 2.1 mm I.D column (Thermo Scientific, USA) using a mobile phase of acetonitrile–ammonium acetate 50 mM (70/30%, v/v) at a flowrate of 0.3 ml min^−1^. The method has been validated according to US FDA guidelines over two calibration ranges 15–720 ng ml^−1^ and 1.5–72 ng ml^−1^.

### 2.11 Statistical analysis of data

Drug dissolution profiles were analyzed using the difference factor (*f*1) and similarity factor (*f*2) as described by Moore and Flanner (1996) and the FDA (1997). Two dissolution profiles were considered similar if *f*1 values were up to 15 and *f*2 values were greater than 50 (Palanisamy and Khanam, 2011, Chevalier et al., 2009).

*In vitro* and *in vivo* data were analyzed using Student bilateral “*t*” test. The difference was considered significant for *p* < 0.05.

## 3 Results and discussion

### 3.1 Development and optimization of AZ suppositories

AZ is a class IV/II BCS drug (WHO, 2005), i.e. exhibiting poor aqueous solubility and/or poor permeability. It remains unclear whether poor bioavailability is due to poor solubility alone, or also poor permeability contributes (WHO, 2005). Solid dispersions, solutions or eutectic mixtures are commonly used to improve the rectal absorption of poorly soluble drugs (Iqbal et al., 2011, Leuner and Dressman, 2000) by enhancing their solubility and dissolution rate (Craig, 2002, Jigar et al., 2011, Kalia and Poddar, 2011), and are compatible with our TPP.

**Fig. 1:**
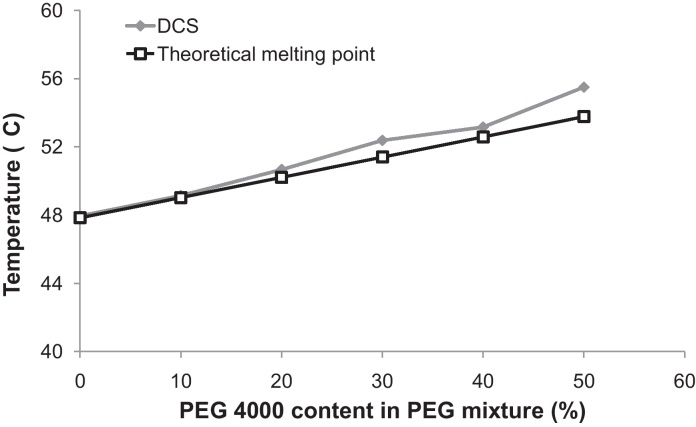
Comparison of theoretical and DSC (differential scanning calorimetry) measured melting point of various mixtures of suppository excipients (PEG 1500 and 4000, expressed as % of PEG 4000 in PEG 4000/1500 mixture). Theoretical melting point was calculated from individual melting points of PEG 1500 and PEG 4000 respecting their relative proportions.

Through previous work in our laboratory, suspended AZ PEG suppositories provided the highest rectal *C*_max_ and AUC_0–24 h_ in rabbits (Kauss et al., 2012) and were selected for further improvement. The bioavailability of AZ suspended suppositories was ∼28% relative to IV Zithromax^®^ – an encouraging result considering the reported 38% oral bioavailability of Zithromax^®^ capsules in humans (Pfizer, 2012). PEGs as hydrophilic base for suppositories present various advantages such as low toxicity, high water solubility and low cost (Parmar et al., 2011), which complied with our TPP. As PEGs might be slightly irritant in children (EMA, 2006), no other surfactants were added to avoid problems with local tolerability or early expulsion of the form. A mixture of two PEGs, one of low and the other of high molecular weight, is commonly used for conventional rapid-release suppositories (Saleem et al., 2008). Different ratios of PEG 1500/4000 were tested; 80% PEG 1500 plus 20% PEG 4000 (melting point >50 °C as per the TPP) was selected (Fig. 1). These results are in line with previous data on PEG, showing that the melting point increases with its molecular weight (Taha et al., 2003, Vippagunta et al., 2007).

AZ could not be dissolved in given proportions in melted PEG mixture at 70 °C. Using ethanol 95% as an intermediate solvent caused AZ to precipitate as soon as ethanol started to evaporate; AZ dissolution in PEG of lower molecular weight (PEG 200, PEG 400) was still incomplete in given proportions after 48 h stirring. The aim of the optimization of AZ suspended suppositories was to obtain a homogenous formulation to accelerate drug release and avoid sedimentation occurring with suspensions. Alternative options to suspended suppositories were: (i) co-melted suppository, as melted AZ (123 °C) and melted PEG mixture (51 °C) were miscible and (ii) solid solution suppository. Suspended, co-melted and solid solution suppositories were prepared with the same quantitative formulation, but through various preparation processes, which lead to different final forms. Table 1 provides information on the formulation of all suppositories and Table 2 on the manufacturing process and characterization.

**Table 1:** Formulation of AZ suppository.

Content	Weight (mg)	Proportion (% w/w)
AZ (equivalent AZ anhydrous)	419 (400)	16.1 (15.4)
PEG 1500	1760	67.7
PEG 4000	440	16.9

**Table 2:** Preparation and *in vitro* characterization of AZ PEG suppository formulations.

Formulation/suppository	Suspended	Co-melted	Solid solution
Preparation			
Process temperature (°C)	65	130	90
Equipment	Water bath	Oven	Water bath (PEG pre-melted)
Process time (min)	64	42	17
*In vitro* characterization			
Suppository aspect	Whitish cloudy	White translucent marbled	White translucent marbled
Melting point	51.1	50	52.2
Melted aspect	Suspension	Cloudy	Crystal clear
Mass			
Mean ± SD (g)	2.56 ± 0.003	2.67 ± 0.064	2.62 ± 0.015
Uniformity ± 5%	Yes	Yes	Yes
Drug content (% of theoretical)	98.3 ± 0.9	97.4 ± 0.6	99.9 ± 0.3
Drug release at 45 min (%)	73.8 ± 1.8*	74.3 ± 2.1	77.3 ± 2.4*
X ray diffraction	AZ crystalline	ND	AZ amorphous
FTIR analysis	No interaction AZ-PEG Hydrogen bond AZ-H_2_O	ND	Interaction AZ-PEG (red shift) Hydrogen bond AZ-H_2_O disrupted (blue shift)

*Key*: ND: not determined.

* *p* = 0.0013 between suspended and solid solution 45 min drug release (*t* test).

Suspended AZ suppositories had the advantage of being prepared at low temperature (65 °C) in a water bath (hence simplicity), but lacked homogeneity and drug release was delayed.

A second option was to mix AZ and PEGs once melted to form co-melted suppositories. However, as the melting point of AZ is high (123 °C), it required the process temperature to be above 125 °C (Table 2), requiring an oven or an oily bath, instead of commonly used water bath. When kept at high temperature over a prolonged period of time, the preparation became cloudy and the colour changed to orange, which was a sign of a degradation of the drug, confirmed by HPLC analysis. Moreover, stability problems have been described for PEGs when manufactured by hot melted method, due to a reduction of PEG chain length (Leuner and Dressman, 2000). Therefore this option was not pursued.

The remaining option was to prepare AZ solid solution suppositories. Solid solutions reportedly increase drug solubility and release (Craig, 2002, Jigar et al., 2011, Kalia and Poddar, 2011) and are simple to manufacture. The optimal process temperature to prepare AZ solid solution suppositories was 80 °C, which represented the best compromise between process time and temperature (the higher the temperature, the shorter the time, but the higher the risks of product degradation). Pre-melting PEGs mixture (2 min at 90 °C) before introducing AZ did not influence the process time and avoided the additional co-grinding step.

### 3.2 *In vitro* characterization of AZ PEG suppositories

Table 2 summarizes the results of *in vitro* characterization of the three types of AZ suppositories, suspended, co-melted and solid solution suppositories. The aim of this evaluation was to compare drug release for solid solution suppositories to the other types. The retention time of AZ by HPLC was 5.7 min. AZ content was within 100 ± 5% drug content for all formulations. Drug released after 45 min was faster for solid solution than suspended suppositories (*p* < 0.01) and statistically not different from co-melted suppositories.

By visual inspection, solid solution AZ suppositories were white, translucent and marbled (same aspect as blank suppositories without AZ), compared to suspended suppositories which were cloudy. When melted, solid solution AZ suppositories were crystal clear the different components are miscible at liquid state, as reported for solid solutions and eutectic mixtures (Leuner and Dressman, 2000). Comparatively, AZ particles in suspension were clearly visible in the suspended AZ suppositories.

**Fig. 2:**
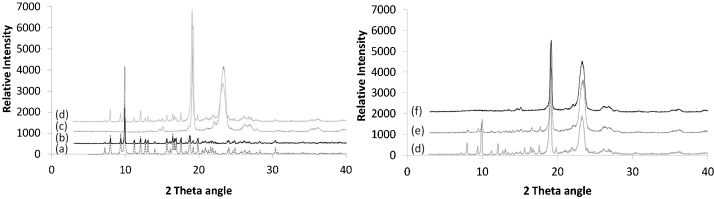
X-ray diffraction patterns of AZ, excipients, physical mixture and the different AZ suppository formulations: crystalline structure of AZ, visible in physical mixture and suspended suppositoryes, disappears in solid solution suppositories. *Key*: (a) theoretical AZ, (b) experimental AZ, (c) excipients, (d) physical mixture, (e) suspended AZ suppository, and (f) solid solution AZ suppository.

**Fig. 3:**
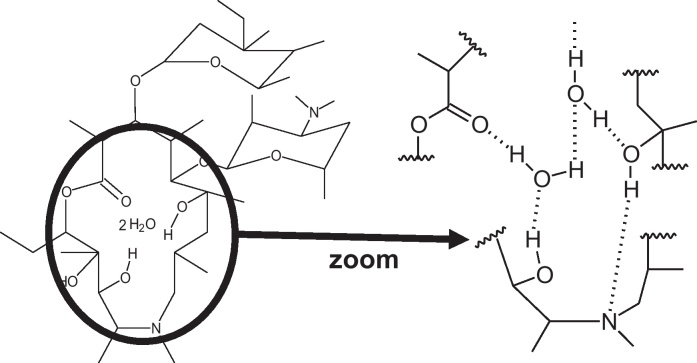
Graphical representation of AZ dihydrate molecular interaction: hydrogen bonds between 2 molecules of crystalline water and polar groups of AZ are important for AZ·2H_2_O spatial arrangement and interfere while solid solution with PEG is formed.

**Fig. 4:**
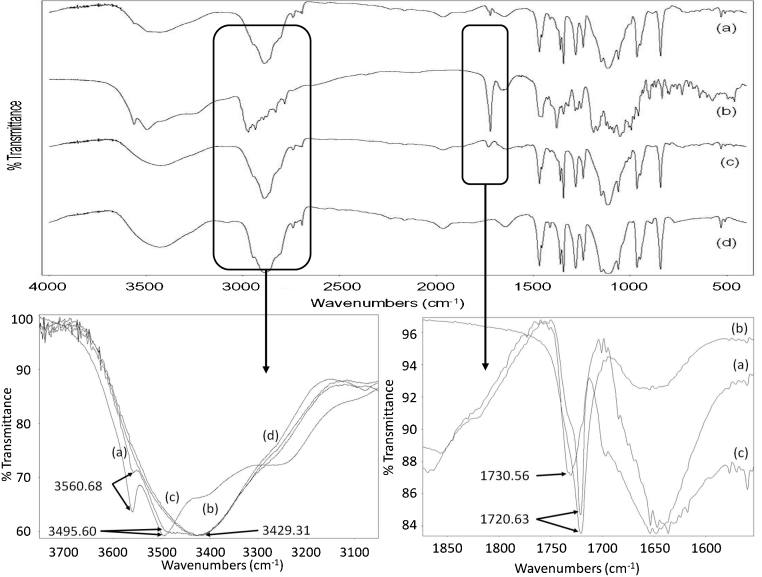
FTIR (Fourier transform infrared spectroscopy) spectra of AZ and blank or AZ containing suppository formulations. *Key*: (a) suspended AZ suppository, (b) AZ, (c) solid solution AZ suppository, and (d) blank (PEG 1500 and 4000) suppository.

DSC was used to determine different melting points and possible interactions between constituents. AZ has a broad melting point peak on DSC thermogram which has been ascribed to the departure of crystalline water simultaneously when melting. Resulting products described are AZ monohydrate and anhydrous azithromycin, reportedly less stable than the dihydrate (Gandhi et al., 2002). DSC thermograms of AZ and PEG 1500/4000 mixture exhibited endothermic melting points at 123.3 °C and 50.7 °C, respectively. On suppository thermograms, the AZ melting peak did not appear. AZ and PEG behaved as a single entity, with one melting point around 51 °C. AZ was not completely soluble in melted PEG at 15.4% (w/w) and precipitated when included in melted PEG as ethanolic solution. A simple dissolution of AZ in PEG could therefore not explain the obtained homogenous preparation. Its lower melting point and rapid drug release of solid solution suppositories favoured a eutectic mixture (Vippagunta et al., 2007), though, the difference in melting points was too narrow to exclude a peritectic system (Sadoway, 2004). The reason for the absence of the endothermic peak of AZ could be that the drug would dissolve and distribute within the melted carrier and convert from crystalline to amorphous form (Damian et al., 2000, Guyot et al., 1995, Khan et al., 2011, Parmar et al., 2011). This conversion is thought to be the reason of the increased solubility and accelerated drug release in eutectic systems and solid solutions (Craig, 2002, Jigar et al., 2011, Kalia and Poddar, 2011) and is confirmed by our drug release results. Furthermore, among the existing methods for solid solution preparation, the fusion method chosen appears to enhance this conversion over solvent method (Jigar et al., 2011). X-ray diffraction performed on solid solution suppositories confirmed the conversion of AZ from crystalline to amorphous form (Fig. 2). The analysis of atomic positions of AZ in its crystalline structure (Fig. 3) showed that both molecules of water were engaged in the spatial arrangement of AZ, and thus are very important for its crystalline pattern. Our findings are consistent with previous reports (Djokic et al., 1988, Gandhi et al., 2002) and with the results of FTIR analysis. Differently from suspended suppository and pure AZ spectra, broader FTIR bands were observed for solid solution suppository, suggesting interactions between components (Fig. 4). In view of the molecular conformation of AZ and the chemical structure of PEG, hydrogen bonds between the free O—H of the components were expected for solid solutions. Indeed, the spectrum presented a red shifting of the O—H stretching (3495.60 and 3560.68 cm^−1^ towards 3429.31 cm^−1^) and a blue shifting of the C=O stretching (1720.63 towards 1730.56 cm^−1^) associated with decreased peak intensity (Fig. 4). These results indicated that one hydrogen bond formed between the free O—H of the AZ and PEG (red shifting) while the second hydrogen bond between a water molecule and AZ was disrupted (blue shifting). These results are consistent with previous data (Ansari et al., 2010) and supported the results of X-ray diffraction.

In conclusion, the *in vitro* characterization indicated that AZ solid solution suppositories gave the fastest drug release and guaranteed optimal physical stability of the formulation (absence of sedimentation), although the exact nature of the solid solution (eutectic or peritectic) remained unclear. This formulation was further evaluated for stability and *in vivo* bioavailability.

### 3.3 Preliminary stability study of AZ solid solution suppositories

Stability is expected to be a limiting factor in the development of solid dispersions (Tajarobi et al., 2011); the pre-stability study of solid solution suppositories was performed before animal experimentation. For each storage condition, namely at ambient temperature/humidity and under accelerated ageing conditions (40 °C/75%RH), two primary conditions, i.e. plastic mould or individual alu/alu blister were tested. Samples were taken at time 0, 2, 5, 8 and 12 weeks to characterize aspect, drug content, *in vitro* drug release and melting point.

No significant change of appearance (solid or melted), melting point or AZ content (100 ± 5%) was noticed during 12-week study for solid solution AZ suppositories in alu/alu blisters (Table 3). For those kept in plastic mould at 40 °C/75%RH, the suppository tail became progressively pastier in few weeks, probably due to water capture. Presence of PEG 1500 in high content is known to make preparations particularly hygroscopic (Price, 2003). The aspect change was paralleled by a decrease in the melting point and a visible peak of water evaporation on the DSC thermogram. Decreased drug content (35% and 7% degradation for suppository tail and head, respectively) was found for AZ plastic mould suppositories after 12 weeks, but not when packaged in alu/alu blisters (Table 3).

**Table 3:** Preliminary stability studies of solid solution AZ suppositories.

	T0	*T* + 12 weeks			
		Ambient condition		40 °C/75%RH	
		Plastic mould	Blister	Plastic mould	Blister
Aspect					
Solid aspect	White/translucent marbled	White/translucent marbled	White/translucent marbled	Head: white/translucent/marbled Tail: pasty/white	White/translucent marbled
Melted aspect	Crystal clear	Crystal clear	Crystal clear	Crystal clear	Crystal clear
Melting point (°C)	52.2	53.3	53.3	Head: 50.0 Tail: 46.3	55.5
Drug content (% of theoretical)	99.9 ± 0.3	101.8 ± 1.9	97.9 ± 0.3	Head: 92.9 ± 2.8 Tail: 64.4 ± 12.0	99.9 ± 0.7
Drug release at 45 min (%)	77.3 ± 2.4	71.8 ± 2.9*	78.2 ± 2.8	69.1 ± 6.7*	75.1 ± 12.3

* *p* < 0.05 (*t* test).

The dissolution profiles of AZ suppositories immediately following their preparation and after 12 weeks were compared using the FDA-defined difference factor *f*1 and similarity factor *f*2 (Chevalier et al., 2009, FDA, 1997, Palanisamy and Khanam, 2011). Results showed that profiles were similar for suppositories from ambient and accelerated ageing conditions when stored in alu/alu blisters, but borderline for similarity (*f*1 = 14.88) for those stored in plastic moulds in accelerated ageing conditions. Drug release after 45 min was significantly modified after 12 weeks (ambient or 40 °C/75%RH) for suppositories kept in plastic moulds (*t*-test, *p* < 0.05), while it remained unchanged when kept in alu/alu blisters.

In conclusion, the preliminary stability study highlighted (i) the good stability of AZ solid solution suppository when kept in alu/alu blister and (ii) the need to protect the suppositories from humidity.

### 3.4 *In vivo* bioavailability study of AZ solid solution suppositories

The plasma profiles obtained in rabbits with AZ solid solution suppositories were compared to rectal suspension used as rectal control (both administered at 20 mg/kg of body weight) and to IV injection (Zithromax^®^) at 10 mg/kg. Doses were determined according to previously published animal pharmacokinetic data (Shepard and Falkner, 1990).

**Fig. 5:**
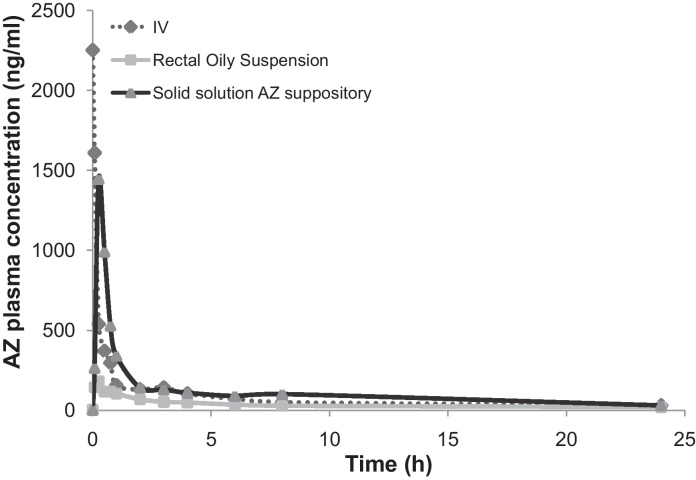
AZ plasma concentration profiles after rectal or IV rabbit administration of AZ at 20 and 10 mg/kg, respectively, in various formulations (IV solution, rectal oily suspension and solid solution suppositories).

The main disposition parameters of AZ are summarized in Table 4 and Fig. 5. Compared to rectal suspension, solid solution AZ suppositories gave an increased *C*_max_, AUC_0–24 h_ and *T*_max_. The estimation of the bioavailability of solid solution AZ suppositories relative to IV was 43% after 48 h (which met our target of 38% – the bioavailability obtained with the oral formulation in humans). Compared to our previous results (Kauss et al., 2012) the rectal and IV references were not statistically different (*p* > 0.05), while the AUC_0–24 h_ increased for the newly formulated solid solution suppository compared to suspended suppository (2697 ng h/ml and 1400 ng h/ml, respectively). The estimation of relative bioavailability against the rectal reference (AUC_0–24 h suppository_/AUC_0–24 h rectal suspension_) was 314% and 232% for solid solution and suspended suppository, respectively. Additionally, *T*_max_ was faster for solid solution compared to suspended suppositories (0.3 h and 1.7 h, respectively) and *C*_max_ was 6 times higher (Fig. 5 and Table 4).

**Table 4:** Pharmacokinetic parameters and comparison of AZ PEG suppositories in rabbits (mean ± S.D.).

Formulation/PK parameters	IV	Rectal suspension	Solid solution suppository	Solid solution/suspended suppository^a^ ratio
*C*_max_ (ng ml^−1^)	2572 ± 1277	185.5 ± 57	1656 ± 334	6.4
*T*_max_ (h)	–	0.183 ± 0.082	0.300 ± 0.100	0.2
AUC_0–24 h_ (ng × h/ml)	2912 ± 911	859 ± 198	2697 ± 819	1.9
Estimated absolute bioavailability 24 h (%)	100	14.7	46.3	1.6
Estimated relative bioavailability 24 h (%)	–	100	314.2	1.4
AUC_0-48h_ (ng × h/ml)	3882 ± 1188	1258 ± 438	3333 ± 1139	ND
Estimated absolute bioavailability 48 h (%)	100	16.2	42.9	ND
Estimated relative bioavailability 48 h (%)	–	100	265	ND

*Key*: ND = not determined.

a Suspended AZ PEG suppository published by Kauss et al. (2012) (data determined during 24 h).

Macrolides are widely distributed in the body, with significant tissue accumulation; they are fat-soluble, basic molecules, which makes them to diffuse easily through biological membranes and accumulate within acidic cellular compartments. These properties could explain the rapid decrease of plasma concentrations during the distribution phase both for IV and solid solution AZ suppository forms (Fig. 5).

In summary, the AZ PEG solid solution suppository shows an improved pharmacokinetic profile, namely better bioavailability (higher *C*_max_ and shorter *T*_max_, which are important for an emergency paediatric treatment), and higher exposure (AUC).

Taking into account the small number (*n* = 5, ethical reasons) of experimental animals, these results are indicative. There was no visible sign of local intolerance. The standard battery of toxicology and toxicokinetic studies will be required if this drug is to be developed further.

### 3.5 Feasibility of solid solution AZ PEG suppositories in view of industrial production scale-up

**Fig. 6:**
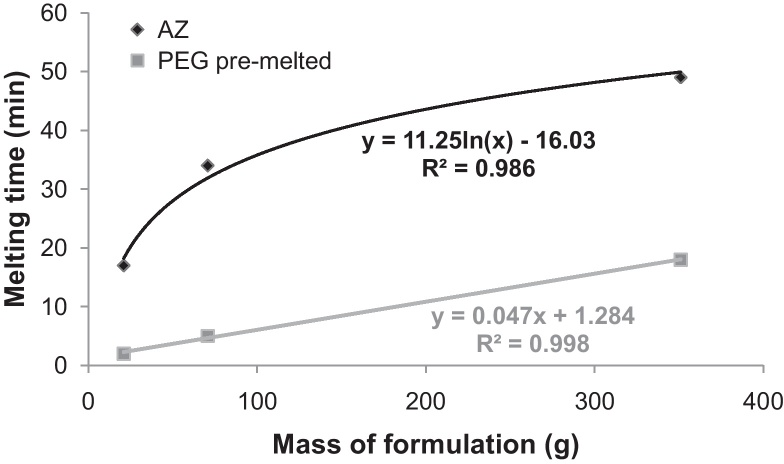
Scale-up modelling for increasing quantity of AZ PEG solid solution suppositories prepared in water bath at 90 °C. Time necessary to obtain homogenous (melted for blank PEG or solid solution for AZ containing) formulation was assessed as a function of the quantity of formulation (size of batch).

The feasibility of scaling-up the production of these AZ PEG suppositories was studied. PEG pre-melting time and AZ-PEG homogenous mixture formation were modelled (see Fig. 6). Linear and logarithmic trend curves with determination coefficients (*R*^2^) were fitted to the experimentally obtained results. For the calculation of the time necessary for pre-melting, the best extrapolation was obtained by linear regression for PEGs and by, logarithmic regression for homogenous AZ PEG mixture.

Product stability formulated under manufacturing conditions (water bath at 90 °C) was determined at 4.5 h: no significant change in AZ HPLC impurity profile was noted; a slight decrease in AZ content (AZ content in 97–106% range from T0.5 h to T3.5 h, but 93% of theoretical content at 4.5 h) concomitant with a slight precipitation occurring between 3.5 and 4.5 h. The process should therefore not exceed 3.5 h in a water bath at 90 °C without further stability studies.

AZ content had been fixed to 400 mg anhydrous AZ, representing 15.4% (w/w) content in suppository formulation (Kauss et al., 2012). In view of future paediatric use, different AZ contents (104–780 mg/suppository) were explored. The formulations containing 104–702 mg of AZ per suppository (i.e. 4.0%, w/w to 27.0%, w/w) gave solid solutions, but precipitation occurred for the drug content higher than 27% (w/w).

The optimized solid solution suppositories provided enhanced rectal bioavailability. With AZ becoming amorphous, drug release was faster and the manufacturing process of homogenous blend easier compared to a suspension. The feasibility of solid solution was only possible under certain conditions (temperature > 80 °C in the presence of PEG), as AZ at defined dose was not soluble in melted PEG at 60 °C. The hygroscopic properties of PEG could explain why the dehydration, which usually occurs above 100 °C (Gandhi et al., 2002), took place at lower temperatures producing monohydrate or anhydrous azithromycin with enhanced solubility in melted PEGs.

## 4 Conclusion

Solid solution AZ suppositories are a candidate product for further development. Optimization work has made the product meet criteria for release, bioavailability, stability and industrial production scale-up. While results must be confirmed in humans, the relative bioavailability in the rabbit of this product (43%) was comparable to that of the oral formulation in humans (38%). This formulation (melting point above 50 °C) can withstand tropical clime (zone IV).

We reckon that this product will have both utility and a potential market. It could complement the oral form for uncomplicated infections and the injectable form for severe infections, but also fill a gap in conditions were oral cannot be taken and injectable is not available or cannot be administered safely. It is easy to administer by unqualified personnel and would be eligible to near-home use.

Furthermore, in addition to its antibacterial effects, AZ possesses antimalarial properties (Andersen et al., 1995, Noedl et al., 2001, Noedl et al., 2007, Yeo and Rieckmann, 1995) and could be used in association with rectal artesunate, an antimalarial drug recommended in “non-per-os” children with malaria (Gomes et al., 2009, WHO, 2010). The advantage of adding AZ would be threefold: mutual protection against malaria parasite resistance; potentially increased activity on malaria; extension of treatment to non-malaria, bacterial infections (acute respiratory infections, sepsis, meningitis) which are currently not covered and are very difficult to distinguish from malaria (Berkley et al., 2005a, Berkley et al., 2005b).

Advancements made in the development of this product with public funds will curtail time and costs of industrial development, thus making the drug affordable.
